# 2-Methyl-3-(2-methyl­phen­yl)-4-oxo-3,4-dihydro­quinazolin-8-yl 4-chloro­benzoate

**DOI:** 10.1107/S1600536812025147

**Published:** 2012-06-13

**Authors:** Adel S. El-Azab, Alaa A.-M. Abdel-Aziz, Amer M. Alanazi, Seik Weng Ng, Edward R. T. Tiekink

**Affiliations:** a Department of Pharmaceutical Chemistry, College of Pharmacy, King Saud University, Riyadh 11451, Saudi Arabia; b Department of Organic Chemistry, Faculty of Pharmacy, Al-Azhar University, Cairo 11884, Egypt; c Department of Medicinal Chemistry, Faculty of Pharmacy, University of Mansoura, Mansoura 35516, Egypt; d Department of Chemistry, University of Malaya, 50603 Kuala Lumpur, Malaysia; e Chemistry Department, Faculty of Science, King Abdulaziz University, PO Box 80203 Jeddah, Saudi Arabia

## Abstract

In the title compound, C_23_H_17_ClN_2_O_3_, the quinazoline fused-ring system, including the ring-bound carbonyl-O and methyl-C atoms, is close to being planar (r.m.s. deviation = 0.044 Å) and is essentially orthogonal to both the 2-tolyl ring [dihedral angle = 89.51 (8)°] and to the ester group [the C—O—C—C torsion angle = −103.69 (16)°]. The carboxyl­ate group is almost coplanar with the benzene ring to which it is attached [O—C—C—C torsion angle = −4.7 (2)°]. The 2-tolyl ring system is disordered over two orientations in a 0.871 (3):0.129 (3) ratio. In the crystal, mol­ecules are consolidated into a three-dimensional architecture by C—H⋯Cl, C—H⋯O, C—H⋯N, C—H⋯π and π–π inter­actions [inter-centroid distances = 3.6443 (9) and 3.8557 (11) Å].

## Related literature

For further synthetic details and the anti-convulsant activity of the title compound, see: El-Azab *et al.* (2011 ▶).
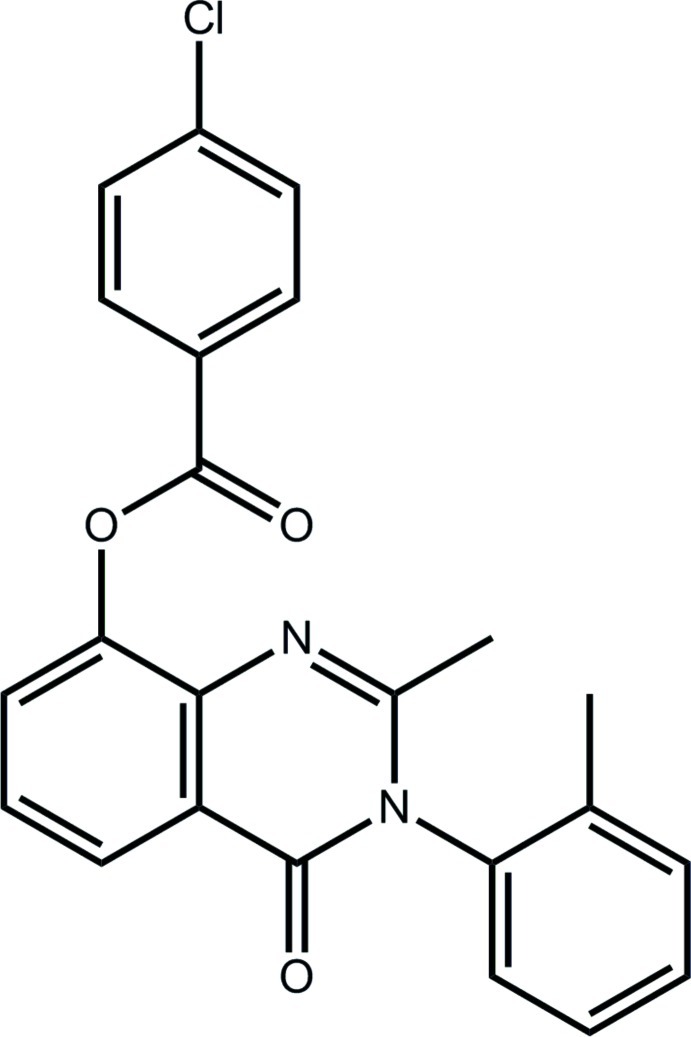


## Experimental

### 

#### Crystal data

C_23_H_17_ClN_2_O_3_
*M* *_r_* = 404.84
Monoclinic, 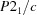

*a* = 18.6703 (5) Å
*b* = 7.6203 (2) Å
*c* = 13.3756 (3) Å
β = 98.006 (3)°
*V* = 1884.44 (8) Å^3^
*Z* = 4
Cu *K*α radiation
μ = 2.03 mm^−1^
*T* = 100 K
0.25 × 0.15 × 0.15 mm

#### Data collection

Agilent SuperNova Dual diffractometer with Atlas detector
Absorption correction: multi-scan (*CrysAlis PRO*; Agilent, 2012 ▶) *T*_min_ = 0.631, *T*_max_ = 0.750
7495 measured reflections
3883 independent reflections
3529 reflections with *I* > 2σ(*I*)
*R*_int_ = 0.020

#### Refinement

*R*[*F* ^2^ > 2σ(*F* ^2^)] = 0.040
*wR*(*F* ^2^) = 0.108
*S* = 1.04
3883 reflections
287 parameters
16 restraints
H-atom parameters constrained
Δρ_max_ = 0.38 e Å^−3^
Δρ_min_ = −0.42 e Å^−3^

### 

Data collection: *CrysAlis PRO* (Agilent, 2012 ▶); cell refinement: *CrysAlis PRO*; data reduction: *CrysAlis PRO*; program(s) used to solve structure: *SHELXS97* (Sheldrick, 2008 ▶); program(s) used to refine structure: *SHELXL97* (Sheldrick, 2008 ▶); molecular graphics: *ORTEP-3* (Farrugia, 1997 ▶) and *DIAMOND* (Brandenburg, 2006 ▶); software used to prepare material for publication: *publCIF* (Westrip, 2010 ▶).

## Supplementary Material

Crystal structure: contains datablock(s) global, I. DOI: 10.1107/S1600536812025147/hb6831sup1.cif

Structure factors: contains datablock(s) I. DOI: 10.1107/S1600536812025147/hb6831Isup2.hkl

Supplementary material file. DOI: 10.1107/S1600536812025147/hb6831Isup3.cml

Additional supplementary materials:  crystallographic information; 3D view; checkCIF report

## Figures and Tables

**Table 1:** Hydrogen-bond geometry (Å, °) *Cg*1 and *Cg*2 are the centroids of the N1,N2,C9–C11,C16 and C1–C6 rings, respectively.

*D*—H⋯*A*	*D*—H	H⋯*A*	*D*⋯*A*	*D*—H⋯*A*
C8—H8*A*⋯Cl1^i^	0.98	2.82	3.6162 (17)	139
C8—H8*C*⋯O2^ii^	0.98	2.51	3.4058 (19)	153
C22—H22⋯N2^iii^	0.95	2.55	3.457 (2)	159
C3—H3⋯*Cg*1^ii^	0.95	2.94	3.834 (2)	158
C12—H12⋯*Cg*2^iv^	0.95	2.81	3.6865 (17)	154

Symmetry codes: (i) 
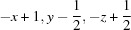
; (ii) 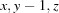; (iii) 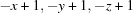; (iv) 
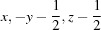
.

**Fig. 1.**
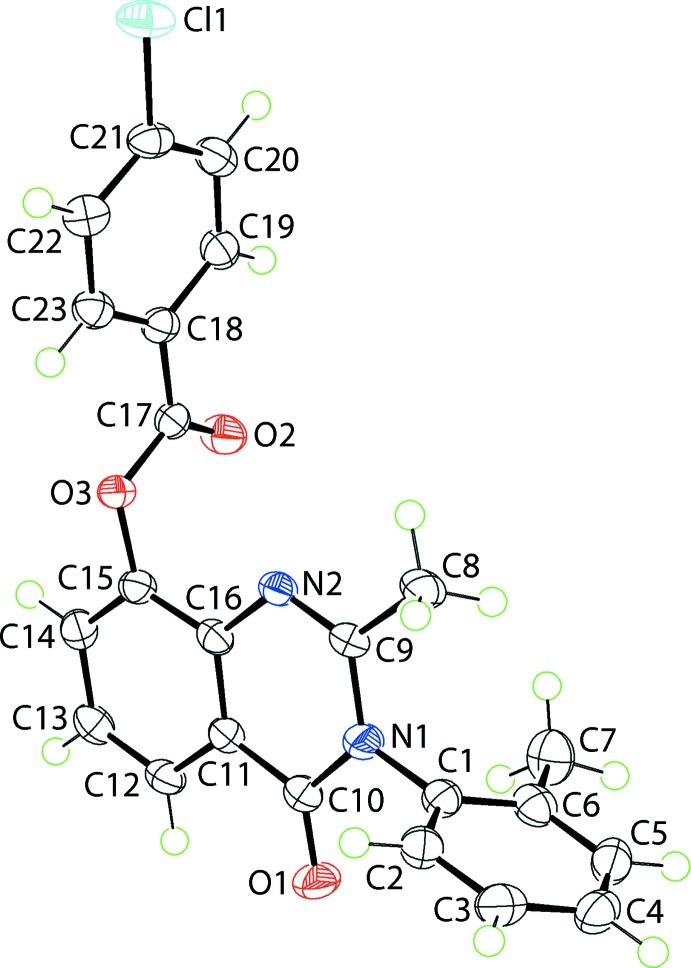
The molecular structure of (I) showing displacement ellipsoids at the 70% probability level.

**Fig. 2.**
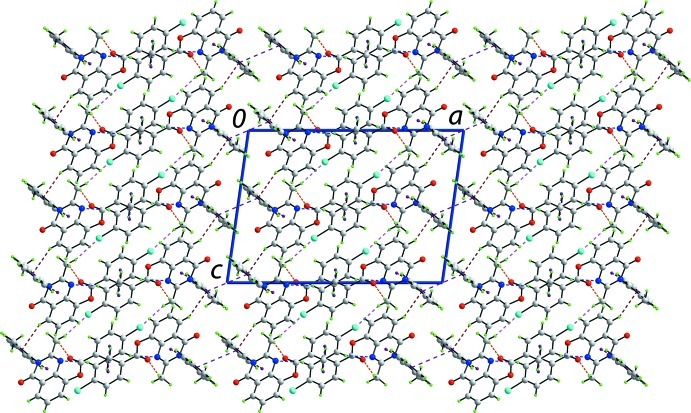
A view in projection down the b axis of the unit-cell contents for (I). The C—H···Cl, C—H···O, C—H···N, C—H···π and π—π interactions are shown as pink, orange, blue, brown and purple dashed lines, respectively.

## supplementary crystallographic information

### Comment

The title compound was previously investigated by us in relation to its biological activity (El-Azab *et al.*, 2011) and we now describe its crystal structure.

The quinazolinyl fused-ring system in (I), Fig. 1, inclusive of the carbonyl-O1 and methyl-C8 atoms, is planar (r.m.s. deviation = 0.044 Å) with maximum deviations of 0.064 (2) Å [C12] and -0.065 (2) Å [C15]. The 2-tolyl ring is orthogonal to this plane, forming a dihedral angle of 89.51 (8)°. The ester group is also twisted significantly out of the plane with the C17—O3—C15—C14 torsion angle being -103.69 (16)°. The carboxylate group is co-planar with the attached benzene ring as seen in the value of the O2—C17—C18—C19 torsion angle of -4.7 (2)°.

Supramolecular layers are formed in the *bc* plane by a combination of C—H···Cl, C—H···O, C—H···N and C—H···π interactions, Table 1, as well as π—π interactions between centrosymmetrically related chlorobenzene rings [inter-centroid distance = 3.6443 (9) Å for symmetry operation: 1 - *x*, 2 - *y*, 1 - *z*]. Links between the layers along the *a* axis are of the type π—π and occur between the major component of the 2-tolyl rings [inter-centroid distance = 3.8557 (11) Å for symmetry operation: -*x*, -*y*, 1 - *z*], Fig. 2.

### Experimental

The compound was prepared in accord with the literature procedure (El-Azab *et al.*, 2011). A mixture of 8-hydroxymethaqualone (532 mg, 0.002 *M*) and 4-chlorobenzoyl chloride (365 mg, 0.0021 *M*) in 10 ml pyridine was stirred at room temperature for 10 h. The solvent was removed under reduced pressure, and the residue was triturated with water and filtered. The solid obtained was dried and recrystallized from EtOH solution as colourless prisms. *M*.pt: 493–495 K. Yield: 95%. ^1^H NMR (CDCl_3_): δ = 8.27–8.08 (m, 3H), 7.61 (d, 1H, J = 7.5 Hz), 7.55–7.37 (m, 6H), 7.15 (d, 1H, J = 7.5 Hz), 2.14 (s, 3H), 2.11 (s, 3H) p.p.m.. ^13^C NMR (CDCl_3_): δ = 17.4, 24.3, 122.5, 125.1, 126.4, 127.1, 127.7, 129.0, 129.4, 131.6, 131.8, 135.4, 136.7, 140.2, 141.4, 146.0, 154.9, 161.1, 164.4 p.p.m.. MS (70 eV): m/*z* = 404, 406 (*M* +2).

### Refinement

Carbon-bound H-atoms were placed in calculated positions [C—H = 0.95 to 0.98 Å, *U*_iso_(H) = 1.2–1.5*U*_eq_(C)] and were included in the refinement in the riding model approximation.

The 2-tolyl group is disordered over two positions in a 0.871 (3): 0.129 (3) ratio. The N—C1 and N—C1' bond lengths were restrained to within 0.01 Å of each other. The 1,2-related C—C distances were restrained to within 0.01 Å and the 1,3-related ones to within 0.02 Å of each other. The anisotropic displacement parameters were restrained to be nearly isotropic and those of the primed atoms were set to those of the unprimed ones.

### Crystal data

**Table:**

C_23_H_17_ClN_2_O_3_	*F*(000) = 840
*M**_r_* = 404.84	*D*_x_ = 1.427 Mg m^−^^3^
Monoclinic, *P*2_1_/*c*	Cu *K*α radiation, λ = 1.54184 Å
Hall symbol: -P 2ybc	Cell parameters from 3710 reflections
*a* = 18.6703 (5) Å	θ = 3.3–76.2°
*b* = 7.6203 (2) Å	µ = 2.03 mm^−^^1^
*c* = 13.3756 (3) Å	*T* = 100 K
β = 98.006 (3)°	Prism, colourless
*V* = 1884.44 (8) Å^3^	0.25 × 0.15 × 0.15 mm
*Z* = 4	

### Data collection

**Table:**

Agilent SuperNova Dual diffractometer with Atlas detector	3883 independent reflections
Radiation source: SuperNova (Cu) X-ray Source	3529 reflections with *I* > 2σ(*I*)
Mirror monochromator	*R*_int_ = 0.020
Detector resolution: 10.4041 pixels mm^-1^	θ_max_ = 76.4°, θ_min_ = 4.8°
ω scan	*h* = −22→23
Absorption correction: multi-scan (*CrysAlis PRO*; Agilent, 2012)	*k* = −9→6
*T*_min_ = 0.631, *T*_max_ = 0.750	*l* = −14→16
7495 measured reflections	

### Refinement

**Table:**

Refinement on *F*^2^	Primary atom site location: structure-invariant direct methods
Least-squares matrix: full	Secondary atom site location: difference Fourier map
*R*[*F*^2^ > 2σ(*F*^2^)] = 0.040	Hydrogen site location: inferred from neighbouring sites
*wR*(*F*^2^) = 0.108	H-atom parameters constrained
*S* = 1.04	*w* = 1/[σ^2^(*F*_o_^2^) + (0.0584*P*)^2^ + 0.9858*P*] where *P* = (*F*_o_^2^ + 2*F*_c_^2^)/3
3883 reflections	(Δ/σ)_max_ = 0.001
287 parameters	Δρ_max_ = 0.38 e Å^−^^3^
16 restraints	Δρ_min_ = −0.42 e Å^−^^3^

### Fractional atomic coordinates and isotropic or equivalent isotropic displacement parameters (Å^2^)

**Table:**

	*x*	*y*	*z*	*U*_iso_*/*U*_eq_	Occ. (<1)
Cl1	0.62417 (2)	0.78020 (5)	0.29279 (3)	0.02700 (13)	
O1	0.10707 (7)	0.32870 (18)	0.65344 (9)	0.0316 (3)	
O2	0.31981 (6)	0.91961 (15)	0.49641 (9)	0.0248 (3)	
O3	0.37759 (6)	0.72310 (14)	0.60522 (8)	0.0183 (2)	
N1	0.17437 (8)	0.3089 (2)	0.52309 (10)	0.0243 (3)	
N2	0.27604 (7)	0.49071 (17)	0.51250 (9)	0.0174 (3)	
C1	0.13094 (9)	0.1590 (2)	0.48158 (13)	0.0196 (4)	0.871 (3)
C2	0.15124 (10)	−0.0104 (2)	0.51140 (14)	0.0216 (4)	0.871 (3)
H2	0.1935	−0.0302	0.5585	0.026*	0.871 (3)
C3	0.10906 (10)	−0.1505 (3)	0.47157 (14)	0.0251 (4)	0.871 (3)
H3	0.1227	−0.2673	0.4905	0.030*	0.871 (3)
C4	0.04682 (11)	−0.1196 (3)	0.40390 (15)	0.0262 (4)	0.871 (3)
H4	0.0176	−0.2151	0.3769	0.031*	0.871 (3)
C5	0.02748 (11)	0.0501 (3)	0.37602 (15)	0.0260 (5)	0.871 (3)
H5	−0.0150	0.0694	0.3293	0.031*	0.871 (3)
C6	0.06855 (10)	0.1944 (2)	0.41446 (13)	0.0223 (4)	0.871 (3)
C7	0.04656 (12)	0.3783 (3)	0.38574 (17)	0.0308 (5)	0.871 (3)
H7A	0.0437	0.4472	0.4469	0.046*	0.871 (3)
H7B	−0.0008	0.3772	0.3437	0.046*	0.871 (3)
H7C	0.0824	0.4308	0.3478	0.046*	0.871 (3)
C1'	0.1078 (5)	0.2358 (13)	0.4647 (8)	0.0196 (4)	0.129
C2'	0.0550 (6)	0.3062 (16)	0.3940 (9)	0.0216 (4)	0.129
H2'	0.0562	0.4281	0.3793	0.026*	0.129 (3)
C3'	0.0004 (6)	0.2041 (13)	0.3439 (9)	0.0251 (4)	0.129
H3'	−0.0368	0.2548	0.2970	0.030*	0.129 (3)
C4'	0.0012 (7)	0.0255 (14)	0.3637 (10)	0.0262 (4)	0.129
H4'	−0.0356	−0.0477	0.3297	0.031*	0.129 (3)
C5'	0.0552 (6)	−0.0469 (15)	0.4329 (9)	0.0260 (5)	0.129
H5'	0.0561	−0.1702	0.4434	0.031*	0.129 (3)
C6'	0.1086 (6)	0.0571 (13)	0.4875 (8)	0.0223 (4)	0.129
C7'	0.1625 (7)	−0.0153 (18)	0.5711 (10)	0.0308 (5)	0.129
H7'1	0.2114	−0.0037	0.5529	0.046*	0.129 (3)
H7'2	0.1520	−0.1394	0.5815	0.046*	0.129 (3)
H7'3	0.1594	0.0501	0.6335	0.046*	0.129 (3)
C8	0.24217 (9)	0.2826 (2)	0.37860 (12)	0.0235 (3)	
H8A	0.2804	0.3440	0.3492	0.035*	
H8B	0.1969	0.2879	0.3317	0.035*	
H8C	0.2561	0.1597	0.3911	0.035*	
C9	0.23159 (8)	0.3684 (2)	0.47614 (11)	0.0194 (3)	
C10	0.15779 (9)	0.3829 (2)	0.61339 (11)	0.0227 (3)	
C11	0.20631 (8)	0.5246 (2)	0.65359 (11)	0.0180 (3)	
C12	0.19532 (8)	0.6111 (2)	0.74303 (11)	0.0200 (3)	
H12	0.1550	0.5818	0.7759	0.024*	
C13	0.24322 (9)	0.7383 (2)	0.78277 (11)	0.0213 (3)	
H13	0.2356	0.7983	0.8426	0.026*	
C14	0.30343 (9)	0.77953 (19)	0.73509 (11)	0.0199 (3)	
H14	0.3370	0.8659	0.7633	0.024*	
C15	0.31367 (8)	0.6948 (2)	0.64760 (11)	0.0179 (3)	
C16	0.26491 (8)	0.5679 (2)	0.60282 (10)	0.0171 (3)	
C17	0.37191 (8)	0.83016 (19)	0.52257 (11)	0.0173 (3)	
C18	0.43716 (8)	0.81991 (19)	0.47050 (11)	0.0175 (3)	
C19	0.43557 (9)	0.9123 (2)	0.38000 (11)	0.0205 (3)	
H19	0.3948	0.9827	0.3558	0.025*	
C20	0.49338 (9)	0.9014 (2)	0.32531 (11)	0.0220 (3)	
H20	0.4925	0.9635	0.2635	0.026*	
C21	0.55220 (9)	0.7988 (2)	0.36223 (12)	0.0208 (3)	
C22	0.55602 (9)	0.7088 (2)	0.45344 (12)	0.0212 (3)	
H22	0.5975	0.6413	0.4783	0.025*	
C23	0.49768 (9)	0.7204 (2)	0.50707 (12)	0.0196 (3)	
H23	0.4991	0.6598	0.5694	0.024*	

### Atomic displacement parameters (Å^2^)

**Table:**

	*U*^11^	*U*^22^	*U*^33^	*U*^12^	*U*^13^	*U*^23^
Cl1	0.0307 (2)	0.0241 (2)	0.0300 (2)	−0.00170 (15)	0.01781 (16)	−0.00279 (15)
O1	0.0292 (6)	0.0445 (7)	0.0237 (6)	−0.0170 (6)	0.0128 (5)	−0.0074 (5)
O2	0.0219 (6)	0.0272 (6)	0.0262 (6)	0.0043 (5)	0.0061 (4)	0.0070 (5)
O3	0.0159 (5)	0.0213 (5)	0.0180 (5)	−0.0016 (4)	0.0036 (4)	0.0032 (4)
N1	0.0256 (7)	0.0319 (7)	0.0167 (6)	−0.0115 (6)	0.0072 (5)	−0.0051 (5)
N2	0.0189 (6)	0.0188 (6)	0.0152 (6)	−0.0011 (5)	0.0049 (5)	0.0009 (5)
C1	0.0204 (9)	0.0219 (10)	0.0172 (8)	−0.0032 (7)	0.0052 (7)	−0.0029 (7)
C2	0.0199 (9)	0.0256 (9)	0.0199 (9)	−0.0003 (7)	0.0042 (7)	−0.0007 (7)
C3	0.0270 (10)	0.0236 (9)	0.0263 (9)	−0.0034 (7)	0.0091 (7)	−0.0006 (7)
C4	0.0250 (10)	0.0303 (10)	0.0245 (9)	−0.0094 (8)	0.0079 (7)	−0.0086 (8)
C5	0.0188 (9)	0.0388 (11)	0.0209 (9)	−0.0035 (8)	0.0043 (7)	−0.0079 (8)
C6	0.0231 (9)	0.0255 (10)	0.0192 (8)	0.0026 (7)	0.0065 (7)	−0.0012 (7)
C7	0.0290 (11)	0.0287 (11)	0.0329 (10)	0.0062 (8)	−0.0020 (8)	0.0016 (9)
C1'	0.0204 (9)	0.0219 (10)	0.0172 (8)	−0.0032 (7)	0.0052 (7)	−0.0029 (7)
C2'	0.0199 (9)	0.0256 (9)	0.0199 (9)	−0.0003 (7)	0.0042 (7)	−0.0007 (7)
C3'	0.0270 (10)	0.0236 (9)	0.0263 (9)	−0.0034 (7)	0.0091 (7)	−0.0006 (7)
C4'	0.0250 (10)	0.0303 (10)	0.0245 (9)	−0.0094 (8)	0.0079 (7)	−0.0086 (8)
C5'	0.0188 (9)	0.0388 (11)	0.0209 (9)	−0.0035 (8)	0.0043 (7)	−0.0079 (8)
C6'	0.0231 (9)	0.0255 (10)	0.0192 (8)	0.0026 (7)	0.0065 (7)	−0.0012 (7)
C7'	0.0290 (11)	0.0287 (11)	0.0329 (10)	0.0062 (8)	−0.0020 (8)	0.0016 (9)
C8	0.0294 (8)	0.0258 (8)	0.0170 (7)	−0.0046 (6)	0.0091 (6)	−0.0032 (6)
C9	0.0207 (7)	0.0236 (7)	0.0148 (6)	−0.0020 (6)	0.0054 (5)	0.0016 (6)
C10	0.0224 (8)	0.0315 (8)	0.0151 (7)	−0.0054 (7)	0.0055 (6)	−0.0017 (6)
C11	0.0182 (7)	0.0210 (7)	0.0147 (6)	−0.0003 (6)	0.0027 (5)	0.0014 (6)
C12	0.0214 (7)	0.0234 (7)	0.0161 (7)	0.0010 (6)	0.0063 (5)	0.0023 (6)
C13	0.0276 (8)	0.0208 (7)	0.0160 (7)	0.0023 (6)	0.0053 (6)	−0.0010 (6)
C14	0.0230 (7)	0.0174 (7)	0.0187 (7)	−0.0009 (6)	0.0005 (6)	0.0003 (6)
C15	0.0167 (7)	0.0193 (7)	0.0177 (7)	0.0000 (6)	0.0031 (5)	0.0036 (6)
C16	0.0185 (7)	0.0186 (7)	0.0144 (6)	0.0018 (6)	0.0027 (5)	0.0026 (5)
C17	0.0189 (7)	0.0160 (6)	0.0169 (6)	−0.0033 (6)	0.0017 (5)	−0.0011 (5)
C18	0.0196 (7)	0.0160 (6)	0.0175 (7)	−0.0038 (6)	0.0039 (5)	−0.0021 (5)
C19	0.0220 (8)	0.0206 (7)	0.0188 (7)	−0.0010 (6)	0.0019 (6)	0.0008 (6)
C20	0.0288 (8)	0.0203 (7)	0.0174 (7)	−0.0042 (6)	0.0054 (6)	0.0003 (6)
C21	0.0235 (8)	0.0188 (7)	0.0221 (7)	−0.0052 (6)	0.0100 (6)	−0.0052 (6)
C22	0.0217 (8)	0.0175 (7)	0.0252 (7)	0.0005 (6)	0.0060 (6)	−0.0001 (6)
C23	0.0219 (7)	0.0180 (7)	0.0193 (7)	−0.0014 (6)	0.0045 (6)	0.0003 (6)

### Geometric parameters (Å, º)

**Table:**

Cl1—C21	1.7424 (16)	C4'—H4'	0.9500
O1—C10	1.223 (2)	C5'—C6'	1.397 (9)
O2—C17	1.1993 (19)	C5'—H5'	0.9500
O3—C17	1.3661 (18)	C6'—C7'	1.502 (9)
O3—C15	1.4075 (18)	C7'—H7'1	0.9800
N1—C9	1.389 (2)	C7'—H7'2	0.9800
N1—C10	1.406 (2)	C7'—H7'3	0.9800
N1—C1	1.465 (2)	C8—C9	1.497 (2)
N1—C1'	1.481 (7)	C8—H8A	0.9800
N2—C9	1.297 (2)	C8—H8B	0.9800
N2—C16	1.3852 (19)	C8—H8C	0.9800
C1—C2	1.388 (3)	C10—C11	1.463 (2)
C1—C6	1.394 (2)	C11—C16	1.405 (2)
C2—C3	1.388 (3)	C11—C12	1.406 (2)
C2—H2	0.9500	C12—C13	1.374 (2)
C3—C4	1.390 (3)	C12—H12	0.9500
C3—H3	0.9500	C13—C14	1.403 (2)
C4—C5	1.380 (3)	C13—H13	0.9500
C4—H4	0.9500	C14—C15	1.373 (2)
C5—C6	1.397 (3)	C14—H14	0.9500
C5—H5	0.9500	C15—C16	1.404 (2)
C6—C7	1.495 (3)	C17—C18	1.487 (2)
C7—H7A	0.9800	C18—C23	1.392 (2)
C7—H7B	0.9800	C18—C19	1.397 (2)
C7—H7C	0.9800	C19—C20	1.388 (2)
C1'—C2'	1.376 (9)	C19—H19	0.9500
C1'—C6'	1.395 (8)	C20—C21	1.382 (2)
C2'—C3'	1.380 (9)	C20—H20	0.9500
C2'—H2'	0.9500	C21—C22	1.393 (2)
C3'—C4'	1.386 (9)	C22—C23	1.388 (2)
C3'—H3'	0.9500	C22—H22	0.9500
C4'—C5'	1.385 (9)	C23—H23	0.9500
			
C17—O3—C15	116.28 (11)	C9—C8—H8B	109.5
C9—N1—C10	122.22 (13)	H8A—C8—H8B	109.5
C9—N1—C1	120.22 (13)	C9—C8—H8C	109.5
C10—N1—C1	117.50 (13)	H8A—C8—H8C	109.5
C9—N1—C1'	121.7 (5)	H8B—C8—H8C	109.5
C10—N1—C1'	109.6 (5)	N2—C9—N1	123.88 (14)
C9—N2—C16	117.81 (13)	N2—C9—C8	119.06 (14)
C2—C1—C6	122.32 (17)	N1—C9—C8	117.06 (14)
C2—C1—N1	120.13 (15)	O1—C10—N1	121.19 (15)
C6—C1—N1	117.53 (16)	O1—C10—C11	124.45 (14)
C3—C2—C1	119.17 (17)	N1—C10—C11	114.36 (13)
C3—C2—H2	120.4	C16—C11—C12	121.14 (14)
C1—C2—H2	120.4	C16—C11—C10	118.66 (13)
C2—C3—C4	119.85 (18)	C12—C11—C10	120.17 (14)
C2—C3—H3	120.1	C13—C12—C11	119.70 (14)
C4—C3—H3	120.1	C13—C12—H12	120.2
C5—C4—C3	119.89 (18)	C11—C12—H12	120.2
C5—C4—H4	120.1	C12—C13—C14	120.11 (14)
C3—C4—H4	120.1	C12—C13—H13	119.9
C4—C5—C6	121.91 (18)	C14—C13—H13	119.9
C4—C5—H5	119.0	C15—C14—C13	119.82 (14)
C6—C5—H5	119.0	C15—C14—H14	120.1
C5—C6—C1	116.85 (17)	C13—C14—H14	120.1
C5—C6—C7	121.74 (18)	C14—C15—C16	121.91 (14)
C1—C6—C7	121.42 (18)	C14—C15—O3	119.85 (13)
C2'—C1'—C6'	121.0 (8)	C16—C15—O3	118.01 (13)
C2'—C1'—N1	133.1 (8)	N2—C16—C15	119.83 (13)
C6'—C1'—N1	105.6 (7)	N2—C16—C11	122.91 (14)
C1'—C2'—C3'	121.6 (9)	C15—C16—C11	117.25 (13)
C1'—C2'—H2'	119.2	O2—C17—O3	122.95 (14)
C3'—C2'—H2'	119.2	O2—C17—C18	125.26 (14)
C2'—C3'—C4'	118.2 (9)	O3—C17—C18	111.79 (12)
C2'—C3'—H3'	120.9	C23—C18—C19	119.83 (14)
C4'—C3'—H3'	120.9	C23—C18—C17	122.81 (13)
C5'—C4'—C3'	120.4 (9)	C19—C18—C17	117.34 (14)
C5'—C4'—H4'	119.8	C20—C19—C18	120.15 (14)
C3'—C4'—H4'	119.8	C20—C19—H19	119.9
C4'—C5'—C6'	121.6 (9)	C18—C19—H19	119.9
C4'—C5'—H5'	119.2	C21—C20—C19	118.87 (14)
C6'—C5'—H5'	119.2	C21—C20—H20	120.6
C5'—C6'—C1'	117.0 (8)	C19—C20—H20	120.6
C5'—C6'—C7'	122.4 (9)	C20—C21—C22	122.20 (15)
C1'—C6'—C7'	120.4 (8)	C20—C21—Cl1	119.27 (12)
C6'—C7'—H7'1	109.5	C22—C21—Cl1	118.53 (13)
C6'—C7'—H7'2	109.5	C23—C22—C21	118.25 (15)
H7'1—C7'—H7'2	109.5	C23—C22—H22	120.9
C6'—C7'—H7'3	109.5	C21—C22—H22	120.9
H7'1—C7'—H7'3	109.5	C22—C23—C18	120.67 (14)
H7'2—C7'—H7'3	109.5	C22—C23—H23	119.7
C9—C8—H8A	109.5	C18—C23—H23	119.7
			
C9—N1—C1—C2	88.8 (2)	C1—N1—C10—O1	−3.0 (3)
C10—N1—C1—C2	−88.4 (2)	C1'—N1—C10—O1	27.8 (4)
C1'—N1—C1—C2	−169.4 (10)	C9—N1—C10—C11	−0.8 (2)
C9—N1—C1—C6	−93.0 (2)	C1—N1—C10—C11	176.30 (14)
C10—N1—C1—C6	89.87 (19)	C1'—N1—C10—C11	−152.9 (4)
C1'—N1—C1—C6	8.9 (10)	O1—C10—C11—C16	176.65 (16)
C6—C1—C2—C3	1.5 (3)	N1—C10—C11—C16	−2.6 (2)
N1—C1—C2—C3	179.62 (16)	O1—C10—C11—C12	−1.4 (3)
C1—C2—C3—C4	−1.0 (3)	N1—C10—C11—C12	179.36 (14)
C2—C3—C4—C5	0.5 (3)	C16—C11—C12—C13	−1.1 (2)
C3—C4—C5—C6	−0.5 (3)	C10—C11—C12—C13	176.87 (14)
C4—C5—C6—C1	0.9 (3)	C11—C12—C13—C14	−1.0 (2)
C4—C5—C6—C7	−178.67 (19)	C12—C13—C14—C15	1.2 (2)
C2—C1—C6—C5	−1.4 (3)	C13—C14—C15—C16	0.8 (2)
N1—C1—C6—C5	−179.61 (15)	C13—C14—C15—O3	−173.65 (13)
C2—C1—C6—C7	178.16 (18)	C17—O3—C15—C14	−103.69 (16)
N1—C1—C6—C7	0.0 (3)	C17—O3—C15—C16	81.68 (16)
C9—N1—C1'—C2'	−63.7 (16)	C9—N2—C16—C15	176.90 (14)
C10—N1—C1'—C2'	88.6 (15)	C9—N2—C16—C11	−2.8 (2)
C1—N1—C1'—C2'	−160 (2)	C14—C15—C16—N2	177.56 (14)
C9—N1—C1'—C6'	110.8 (8)	O3—C15—C16—N2	−7.9 (2)
C10—N1—C1'—C6'	−96.9 (9)	C14—C15—C16—C11	−2.7 (2)
C1—N1—C1'—C6'	14.7 (6)	O3—C15—C16—C11	171.76 (12)
C6'—C1'—C2'—C3'	1 (2)	C12—C11—C16—N2	−177.41 (14)
N1—C1'—C2'—C3'	174.7 (12)	C10—C11—C16—N2	4.6 (2)
C1'—C2'—C3'—C4'	−2 (2)	C12—C11—C16—C15	2.9 (2)
C2'—C3'—C4'—C5'	1 (2)	C10—C11—C16—C15	−175.09 (13)
C3'—C4'—C5'—C6'	3 (2)	C15—O3—C17—O2	12.3 (2)
C4'—C5'—C6'—C1'	−4 (2)	C15—O3—C17—C18	−167.29 (12)
C4'—C5'—C6'—C7'	172.8 (14)	O2—C17—C18—C23	176.69 (15)
C2'—C1'—C6'—C5'	2.3 (19)	O3—C17—C18—C23	−3.7 (2)
N1—C1'—C6'—C5'	−173.0 (10)	O2—C17—C18—C19	−4.7 (2)
C2'—C1'—C6'—C7'	−174.6 (13)	O3—C17—C18—C19	174.85 (13)
N1—C1'—C6'—C7'	10.0 (16)	C23—C18—C19—C20	1.6 (2)
C16—N2—C9—N1	−1.0 (2)	C17—C18—C19—C20	−176.97 (13)
C16—N2—C9—C8	179.94 (13)	C18—C19—C20—C21	−0.3 (2)
C10—N1—C9—N2	2.8 (3)	C19—C20—C21—C22	−1.3 (2)
C1—N1—C9—N2	−174.24 (15)	C19—C20—C21—Cl1	178.53 (12)
C1'—N1—C9—N2	151.6 (5)	C20—C21—C22—C23	1.5 (2)
C10—N1—C9—C8	−178.10 (15)	Cl1—C21—C22—C23	−178.29 (11)
C1—N1—C9—C8	4.9 (2)	C21—C22—C23—C18	−0.2 (2)
C1'—N1—C9—C8	−29.3 (5)	C19—C18—C23—C22	−1.4 (2)
C9—N1—C10—O1	179.89 (16)	C17—C18—C23—C22	177.15 (14)

### Hydrogen-bond geometry (Å, º)

Cg1 and Cg2 are the centroids of the N1,N2,C9–C11,C16 and C1–C6 rings, respectively.

**Table:**

*D*—H···*A*	*D*—H	H···*A*	*D*···*A*	*D*—H···*A*
C8—H8*A*···Cl1^i^	0.98	2.82	3.6162 (17)	139
C8—H8*C*···O2^ii^	0.98	2.51	3.4058 (19)	153
C22—H22···N2^iii^	0.95	2.55	3.457 (2)	159
C3—H3···*Cg*1^ii^	0.95	2.94	3.834 (2)	158
C12—H12···*Cg*2^iv^	0.95	2.81	3.6865 (17)	154

Symmetry codes: (i) −*x*+1, *y*−1/2, −*z*+1/2; (ii) *x*, *y*−1, *z*; (iii) −*x*+1, −*y*+1, −*z*+1; (iv) *x*, −*y*−1/2, *z*−1/2.
